# Nanomaterials for Membranes, Membrane Reactors, and Catalyst Systems

**DOI:** 10.3390/nano12060964

**Published:** 2022-03-14

**Authors:** Gheorghe Nechifor

**Affiliations:** Analytical Chemistry and Environmental Engineering Department, University Politehnica of Bucharest, 011061 Bucharest, Romania; gheorghe.nechifor@upb.ro

Membranes are selective and highly productive nanostructures dedicated to developing separation, concentration, and purification processes with uses in the most diverse economic and social fields: industry, agriculture, transport, environment, health, and space exploration. On the other hand, the sensitivity and catalytic activity of membranes have widened their applicability to the most complex environmental, biotechnological, biomedical, and technological analyses.

This Special Issue aims to offer readers a compilation of cutting-edge research of nanomaterials’ impact on membranes, membrane reactors, and catalyst systems.

The use of nanomaterials in the realization of membranes and processes based on them has brought important benefits to users, such as increasing physical performance—mechanical, thermal, electrical, or magnetic; improving chemical performance—pH, redox, ion exchange, and complexation; amplification of activity and sensitivity—catalysis, bio-catalysis, sensors, and detectors; development of biological characteristics—biocompatibility, biodegradability, anti-biofouling, and guided transport [1].

Some of these advantages of using nanomaterials in membrane technologies have been developed in this Special Issue.

Thus, the sensitivity and selectivity of stochastic electrochemical sensors have been shown to be amplified using nano-films and composite nanoparticles in biomedical applications [2].

On the other hand, advanced electrochemical methods allow controlling the formation of membrane structures dedicated to protein separation [3].

Affordable magnetic composite nanoparticles enable direct transport through supported liquid membranes [4] and the removal of potentially toxic organic compounds from aqueous media [4,5].

Nanoparticle engineering ensures the advantageous recovery of various wastes [1] and their use in catalytic, photocatalytic, and separative processes of special technical interest [1,6].

The nanoencapsulation of natural extracts and biomedical preparations is illustrated by a current example of drug delivery [7].

The obtainment and application of nanomaterials in the form of the film have found excellent uses of great technological utility [8,9,10], even reaching the needs of aerospace and advanced manufacturing technology [11].

Readers will certainly find other interesting aspects of the implications of nanomaterials in membranology by studying the papers presented in this Special Issue of “Nanomaterials for Membranes, Membrane Reactors, and Catalyst Systems”.

We hope that all readers will enjoy it.
